# Lessons from mouse models of Graves’ disease

**DOI:** 10.1007/s12020-020-02311-7

**Published:** 2020-05-12

**Authors:** A. Eckstein, S. Philipp, G. Goertz, J. P. Banga, U. Berchner-Pfannschmidt

**Affiliations:** 1 Department of Ophthalmology, Medical Faculty, University Duisburg-Essen, Essen, Germany; 2 Laboratory of Molecular Ophthalmology, Medical Faculty, University Duisburg-Essen, Essen, Germany; 3 Emeritus Professor, Faculty of Life Sciences & Medicine, King’s College London, London, UK

## Abstract

Graves’ disease (GD) is an autoimmune condition with the appearance of anti-TSH receptor (TSHR) autoantibodies in the serum. The consequence is the development of hyperthyroidism in most of the patients. In addition, in the most severe cases, patients can develop orbitopathy (GO), achropachy and dermopathy. The central role of the TSHR for the disease pathology has been well accepted. Therefore immunization against the TSHR is pivotal for the creation of in vivo models for the disease. However, TSHR is well preserved among the species and therefore the immune system is highly tolerant. Many differing attempts have been performed to break tolerance and to create a proper animal model in the last decades. The most successful have been achieved by introducing the human TSHR extracellular domain into the body, either by injection of plasmid or adenoviruses. Currently available models develop the whole spectrum of Graves’ disease—autoimmune thyroid disease and orbitopathy and are suitable to study disease pathogenesis and to perform treatment studies. In recent publications new immunomodulatory therapies have been assessed and also diseaseprevention by inducing tolerance using small cyclic peptides from the antigenic region of the extracellular subunit of the TSHR.

## What is Graves’ disease?/Pathogenic mechanism of Graves’ disease

Autoimmunity to the thyroid-stimulating hormone receptor (TSHR) plays the central role for the pathogenesis of Graves’ disease (GD) [1]. Binding of stimulating anti-TSH-receptor antibodies (TRAb) leads to hyperthyroidism, which is uncontrolled by the pituitary [2]. The consequences of increased production of thyroid hormones are manifold: weight loss, tachycardia, hyperthermia to name only a few. The TSH receptor is also expressed by orbital fibroblasts [3]. Binding of TRAb to orbital fibroblasts leads to hyaluronan production and differentiation to adipocytes and myofibroblasts [4–6]. The consequence is the increase of orbital fat and also fibrosis of the orbital connective tissues, especially the extraocular muscles. In the confines of the bony orbit, this leads to proptosis, swelling of the soft tissues and to restricted eye movements with diplopia [7]. The stimulation of the TSHR on the orbital fibroblasts leads to a pathologic cross talk between the TSHR and the Insulin-like growth factor 1 receptor [8–10], which plays an important role for the stimulation of all the processes induced.

Besides the proliferative stimulation of the orbital fibroblasts, inflammatory cytokines are released which act to recruit additional immune and inflammatory cells to the orbit [11, 12]. The CD40 expression of orbital fibroblasts allows direct interaction with infiltrating T cells with potential additional cytokine release [13]. Since the inflammatory/proliferative processes take place in a bony limited space—the orbit—tissue hypoxia contributes to the pathogenic mechanisms depending on the grade of compression which is caused by the tissue volume changes [14].

Due to many influencing factors genetic and environmental Graves’ disease has highly variable phenotypes and time relations between the onset of thyroid and eye disease.

## Induction of an animal model for Graves’ disease—break tolerance to TSHR

The development of animal models for Graves’ disease were accelerated with the sequencing of the TSHR and consequently the availability of recombinant TSHR DNA, which made it amenable to prepare substantial quantities of recombinant TSHR protein or synthetic peptides for active immunization. In addition, the development of innovative techniques leading to in vivo expression of the receptor was the key to successful models of Graves’ disease [15].

**Fig. 1:**
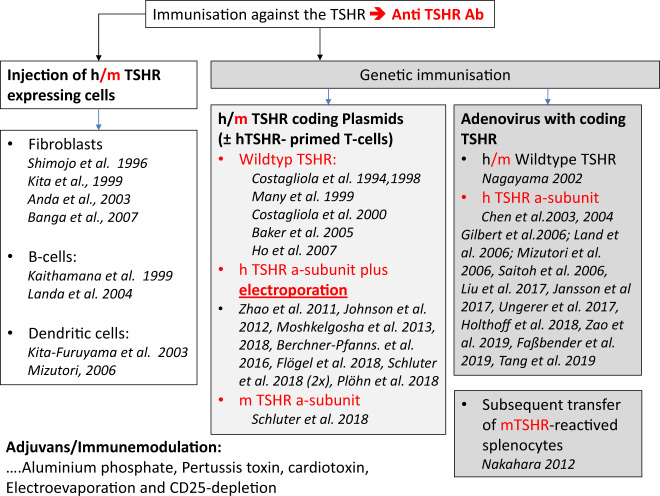
Immunization against the TSHR was realized by inducing in vivo-expression of the TSHR by using different approaches

Immunization against the TSHR was realized by inducing in vivo expression of the TSHR by using different approaches (see Fig. 1): (1) Injection of TSHR-expressing cells, (2) genetic immunisation using TSHR-expressing adenovirus, and (3) genetic immunisation by using plasmids encoding for the TSHR. Injection of TSHR-expressing cells delivered less solid induction of autoimmunity and was therefore abandoned—summary in [16].

Nagayama et al. described a mouse model of Graves’ disease that is based on genetic immunization by TSHR-adenovirus and was tested on different strains of inbred mice [17]. Female BALB/c mice reached the highest disease rates. Based on the observation of Chazenbalk et al. [18] that TRAbs in Graves’ disease preferentially recognize the free A subunit of the TSHR. Chen et al. [19] modified the adenovirus model concerning the antigen. It was shown that immunization by Adenovirus-TSHR289, which encoded only the A subunit of the human TSHR-induced hyperthyroidism and TRAb to a greater extent than immunization by adenovirus carrying the full length TSHR wild type. Incidence of hyperthyroidism was reported to be up to 86%. This model has been shown to be highly reproducible—summary in [16] and is widely used. Several modifications have been developed like additional depletion of CD25+ T cells [20].

Genetic immunization by using plasmids encoding the TSHR also has a long history of use and was first published by investigators from Brussels, who were also able to demonstrate some disease transfer using TSHR-primed splenocytes [21–23]. Different plasmids, even encoding TSHR genes with activating mutations [24] have been used. However, as with protocols using adenovirus immunization, the success for genetic immunization with plasmids was greatly improved by employing the TSHR-A-subunit, rather than the holoreceptor, and a special technique electroporation [25]. This approach resulted in a high incidence of induced hyperthyroidism accompanied by a sustained antibody response to TSHR. Importantly, histological investigation of orbital tissue gave the first indication of orbital inflammation and fibrosis in the model [26]. The model is highly reproducible and the pathological features of orbital remodeling were more reminiscent of those present in Graves’ orbitopathy (GO) patients [27–29].

Today there are two potent techniques for inducing in vivo models of GD applied—adenoviruses and plasmids—ready to do extensive studies on pathogenesis to identify new targets for therapy and to perform treatment studies.

## Actual pathogenetic studies with GD/GO in in vivo models

To follow inflammation and remodeling of the orbital tissue in a noninvasive manner in vivo, we have established a powerful imaging strategy with a 9.4 T micro imaging system. Beyond anatomical hydrogen-1 (1H) MRI, we employed transverse relaxation time (T2) mapping for visualization of oedema, chemical exchange saturation transfer for detection of hyaluronan, and fluorine-19 MRI for tracking of in situ-labeled immune cells after intravenous injection of perfluorcarbons. Fluorine-19 MRI-based visualization of orbital inflammation exhibited the highest significance level to discriminate between GO and control mice and showed the best correlation with the clinical score [28].

The induced Graves’ disease displays a variable phenotype, as occurs in GD patients It is similar in the in vivo model. It is therefore reasonable to test the influence of environmental factors on the disease phenotypes.

It is now widely known that the gut microbiota has considerable influence on our immune system.

Under the hypothesis that gut microbiota may modulate clinical presentation of disease a two center study was performed. Variations in a TSHR-induced model of GD/GO in 2 independent centres, correlated with gut-microbiota α and β diversity. A significant positive correlation between the presence of Firmicutes and orbital-adipogenesis specifically in TSHR-immunized mice was observed [30].

To investigate further, in subsequent experiments we modified the gut microbiota of the female BALB/c mice using antibiotic vancomycin, probiotic or human (from patients with GO) faecal material transfer prior to immunization. The reduced thyroid and orbital pathology, combined with diminished lymph node CD25+ T cells, in vancomycin treated mice support a role for the gut microbiota in promoting GO [26].

It appears that these findings contrast an earlier study, in which depletion of CD25+ T cells increased disease susceptibility [20]. The two studies may indicate that relative changes in CD25+ T-cell numbers (either depletion or reduction) can have different consequences. However, we cannot exclude that the relative CD25+ T-cell numbers declined during the antibiotic treatment as a result of an ongoing immune suppression. Moreover, the studies only report the percentage of CD25+ T cells and did not access functional activity. It is known that CD25+ T cells exert regulatory functions but can also (re)gain effector functions. However, it is well accepted that T-cell populations expressing the transcription factor forkhead box protein 3 (Foxp3) constitute T-cell lineages with suppressive functions [31, 32]. With respect to this, our study is limited since it did not investigate Treg populations expressing Foxp3 which could have been differently affected by the antibiotic treatment. A few studies addressed the role of Treg expressing FoxP3 in GD/GO mouse models [33–36]. In a GD mouse model induced by adenovirus Ad-TSHR289, the frequencies of CD4+CD25+Foxp3+ Treg cells were decreased [34, 36]. More recently, we modulated T-cell proportions in our GD/GO model by treatment with the immunomodulatory drug fingolimod. Of note, the suppression of TSHR autoimmunity was accompanied by a decrease in CD25+ T cells while subsets CD25+Foxp3+ Tregs were increased [35]. Whether the observed changes in Treg proportions represent their role in improving TSHR autoimmunity remains to be proven by further studies addressing the functional activity of the different T-cell subsets in the model.

Another group studied the influence of 5α-dihydrotestosterone (DHT) a potent bioactive androgen in the mouse model. It could be shown that DHT can alleviate the severity of GD by downregulating pro-autoimmune T helper 1 cells in female BALB/c mice. The protective influence was dose dependant [37]. In a follow-up study it could be shown that DHT acts on cytokines and oxidative stress markers. Thyroid hormones were significantly reduced in DHT treated GD mice in comparison to the untreated control group. In addition, DHT attenuated thyroid oxidative injuries in GD mice, as shown by decreased total antioxidation capability (TAOC), superoxide dismutase (SOD) and the level of malondialdehyde (MDA). The levels of immunosuppressive cytokines (TGF-β, IL-35) in DHT group were significant higher compared with the control group [35].

## Treatment studies with in vivo models of GO—translation into the clinic

Recently, a few trails testing immunomodulatory strategies for treatment of GD have been published.

One of the tested therapeutic approaches was to administer small amounts of antigens over time to reinstates immune tolerance. Ungerer et al. aimed to induce B- and T-cell anergy by using long and short cyclic peptides derived from the first and eighth cylindrical loops of the leucin-rich repeat domain of the TSHR. The peptides were tested in a mouse model of Graves’ disease where TSHR autoimmunity was induced by long-term protocol of four-weekly immunizations with adenovirus coding for the TSHR A-subunit (Ad-TSHR289). The short cyclic peptides from the first leucin-rich repeat loop turned out to be most efficient in reducing thyroid size, serum thyroxine levels, retro-orbital fibrosis, and tachycardia. In immunologically naïve mice, administration of the peptides did not induce any immune response.

Therefore, cyclic peptides may provide an additional therapeutic option compared to existing drugs or interventions [38, 39].

Another group attempted tolerance induction with the use of synthetic peptides that mimic naturally processed CD4 T-cell epitopes termed “apitopes,” short for antigen-processing independent epitopes. The CD4 T-cell apitopes from TSHR were first tested in an HLA-DR3 transgenic mice and in the Ad-TSHR289 mouse model. The apitopes were able to reduce TSHR-induced proliferation of splenocytes in HLA-DR3 transgenic mice.

In the Ad-TSHR289 mouse model a preventive treatment schedule was applied meaning application of two different mixtures of immunodominant apitopes before the induction of autoimmunity with the Ad-TSHR289. Both mixtures of apitopes were able to suppress TSHR autoimmunity antibody production and reduction of thyroid hormone levels [40].

The animal study was followed by a phase I human trial with the most efficient mixture of apitopes ATX-GD-59. ATX-GD-59 was given in a dose escalation protocol in the first 8 weeks and in full dose another 10 weeks. Patient with untreated mild-to-moderately severe Graves’ hyperthyroidism were included in the study. Levels of free thyroid hormones decreased in 70% of subjects receiving the medication and ATX-GD-59 was safe and well tolerated. Further studies are needed for this novel treatment rational for Graves’ hyperthyroidism [41].

Tolerance induction was not disrupted by current drug treatments. These results demonstrate that antigen-specific immunotherapy with apitopes from TSHR is a suitable approach for treatment of GD [40].

Another recent in vitro study suggested that sphingosine-1-phosphate (S1P) signaling is involved in orbitopathy [13]. In a follow-up study the immune modulatory potential of S1P receptor antagonist fingolimod (FTY720) was explored in a murine model for Graves’ disease [33]. In the model TSHR autoimmunity was induced by immunization with a plasmid encoding for the A-subunit of the TSHR. Fingolimod was orally administered preventively during disease onset or therapeutically after disease onset. Administration of fingolimod during disease onset completely prevented the formation of TSHR-stimulating autoantibodies. Intervention after disease onset rarely reduced TSHR-stimulating autoantibodies. However these therapeutically treated animals showed milder manifestation than the animals in the untreated control group. Clinically they showed less increase of body temperature, weight gain and tachycardia. In mice weight gain is a sign of hyperthyroidism most likely caused by a higher food intake by the hyperthyroid mice [42].

The therapeutically treated mice developed less often overt autoimmune hyperthyroidism characterized by less elevated serum thyroxine levels, less hyperplastic thyroid morphology accompanied by T-cell infiltration.

Importantly, examination of orbital tissue showed significant amelioration of orbitopathy manifestations by reduction of T-cell infiltration, adipogenesis and hyaluronan deposition [35].

**Fig. 2:**
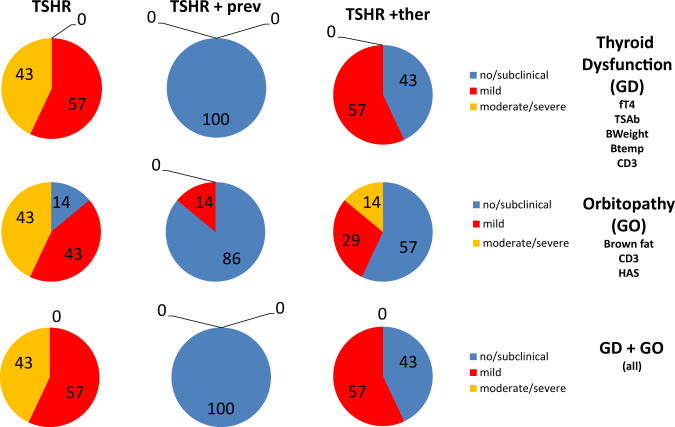
Treatment with fingolimod in a preclinical mouse model: animals were treated with the first immunisation during disease onset (preventive treatment; TSHR +prev) or after the last immunisation with TSHR extracellular coding plasmid (therapy in the state of fully developed disease; TSHR +ther). Results of different parameters were combined and analyzed by Z-scoring for autoimmune hyperthyroidism (GD—including results for fT4, TSAb [stimulating anti-TSH-receptor antibodies], bodyweight, body temperature, CD3 infiltration of the thyroid), orbitopathy (GO—including brown fat content, CD3 infiltration in the orbital tissues, Hyaluronan acid content) or total disease (GD + GO). Percentages of mice that have developed either no (*Z*-score < 0, blue), mild (*Z*-score > 0 but < 1, red) or moderate/severe (*Z*-score > 1, yellow) disease are indicated

Analysis of total disease outcome revealed that treatment during disease onset protected animals against autoimmune hyperthyroidism and orbitopathy. Of note, therapeutic intervention after disease onset suppressed disease in half of the animals and in the other half disease remained at mild stages (see Fig. 2). Based on the results of that study a clinical trial to investigate the immunologic and clinical benefits of early treatment with S1P-based drugs in Graves’ disease can be suggested [33].

## Conclusion

The development of an animal model for Graves’ disease is dependent on the immunization against the human TSHR, providing compelling evidence for the TSHR abeing the primary autoantigen of the disease. Tolerance against the evolutionary conserved TSHR can be broken by introducing plasmids or adenoviruses encoding for the TSHR extracellular into the animal body. The currently available models develop the whole spectrum of Graves’ disease—autoimmune thyroid disease and orbitopathy. A difficulty is the variable clinical phenotype, which is found in humans as well as in the animal models, making higher numbers of animals per experiment necessary. However, the similarity between human disease and the animal model allows the study of disease pathogenesis, the natural history of the disease and events that underlie the continued progression of the autoimmune response to the orbital components in GO. Moreover, most important the animal models are suitable to perform treatment studies.

In one of the treatment studies, fingolimod has been used which causes the internalization of S1P receptors, which sequesters lymphocytes in lymph nodes, preventing them from moving to sites of inflammation. Fingolimod has been shown to prevent the disease completely and it is also therapeutically efficient in overt Graves’ orbitopathy/hyperthyroidism. Another trial addressed tolerance induction by using small cyclic peptides from the antigenic region of the extracellular subunit of the TSHR. The administration of these cyclic peptides reduced hyperthyroidism and orbital manifestations. Research to test the effects of the modification of the GUT microbiota are only at the beginning. Further studies are eagerly awaited.
